# Acute Effects of Carbon Fiber Insole on Three Aspects of Sports Performance, Lower Extremity Muscle Activity, and Subjective Comfort

**DOI:** 10.3390/s23042154

**Published:** 2023-02-14

**Authors:** Myeonghoon Ko, Tiejun Ma, Shuping Xiong

**Affiliations:** Department of Industrial and Systems Engineering, Korea Advanced Institute of Science and Technology (KAIST), Daejeon 34141, Republic of Korea

**Keywords:** footwear, carbon fiber insole, sports performance, comfort, muscle activation

## Abstract

Carbon fiber insole (CFI), which is lightweight and stiff to reduce energy loss and help wearers perform better in sports, has recently been introduced. However, reports are scarce on the effects of CFI on sports performance, muscle activation, and wearing comfort. This study investigated the acute effects of CFI on sports performance, lower extremity muscle activity, and subjective comfort. Thirty young healthy males with shoe sizes between 260 and 270 mm performed various sports tasks (power generation, agility, and speed) and treadmill runs with wearable sensors under two experimental insole conditions (benchmark insole as a baseline, CFI). The results showed that, compared to the benchmark insole, CFI significantly improved sports performance in terms of power generation (~1.5%) and agility (~1%). However, it activated more of the Tibialis Anterior (~0.7%) and Gastrocnemius Medialis (~0.8%) muscles, and was perceived to be stiffer and less comfortable. These findings suggested that CFI could improve sports performance, but could cause more lower extremity muscle activation and subjective discomfort.

## 1. Introduction

Wearing the proper athletic footwear is essential for an athlete to improve comfort, prevent injury, and most importantly, enhance athletic performance [1]. As a result, the impact of footwear on athletic performance has been extensively studied, especially from an energy perspective [2,3]. During athletic movements, there are two phases: one where energy is absorbed and the other where energy is generated. Thus, the main strategy to improve sports performance is to increase energy return and reduce energy loss [4,5]. Although many attempts have been made to effectively return energy, few have been successful because strong conditions must be satisfied at the same time: the energy must return at the correct location, at the right moment, and with the precise frequency [2,6,7]. As a result, there has been extensive research on minimizing the loss of energy during dynamic activities. If the energy is absorbed and dissipated or not stored for later use, it will be inefficient for performing sports activities. Therefore, a reduction in energy absorption may lead to an increase in athletic performance. Especially, the metatarsophalangeal (MTP) joint was predominantly dorsiflexed during the stance phase, resulting in negative work and no energy generation before take-off [8,9]. Consequently, a way of limiting the range of motion to reduce energy loss in the MTP joint was proposed, and it was confirmed that this could be achieved by stiffening the footwear, such as by embedding a carbon plate in the shoes.

Footwear could play an important role in inducing localized pain, muscular activation and fatigue, and wearing discomfort, all of which can limit sports performance. Effects on muscular fatigue and wearing discomfort have been reported according to the different features and characteristics of the insole, such as material, thickness, and wedge for arch support [10,11,12]. Especially, insoles composed of soft material were more effective in creating a more uniform, less localized plantar pressure, and lower fatigue index [13,14]. However, more rigid and stiffer insoles demand more muscle work to absorb impact or provide greater propulsive force to push it, which could result in muscular fatigue and wearing discomfort [1,15,16,17,18]. Additionally, advanced footwear with a full-length stiff plate embedded showed a greater increase in positive work, which implies increased positive work from active foot muscle contractions [19]. The point of the force application was also affected by the increased footwear stiffness. Stiffer footwear showed the increased moment arm of the ground reaction force, which could increase the ankle joint moment and the force demand [20]. Since the triceps surae muscle group containing the soleus, lateral gastrocnemius, and medial gastrocnemius is primarily responsible for absorbing and generating the power of the ankle joint, the use of rigid footwear could induce more activation of these muscles.

Several studies have explored the role of carbon plates embedded in shoes on sports performance and injury. They have reported that stiffer shoes improve running economy, speed, agility, and jump performance [21,22,23,24]. As the different combinations of shoe designs and carbon plates may affect the results due to confounding, it is difficult to examine the direct effect of carbon fiber plates. Only a few studies have examined the effects of insoles with carbon fiber plates. Gregory et al. studied the effects of carbon fiber insoles (CFI) on athletic performance, and they reported that CFI could help athletes perform better by minimizing energy loss [25]. Furthermore, it could increase the ratio of the lever arms of the output ground reaction force and the lever arm of the ankle plantar flexor, termed the gear ratio, by adding extra gear to the foot [26]. A higher gear ratio could induce a slower shortening velocity of the plantar flexor muscles, which could improve force production on account of the force-velocity relationship [27,28]. Thus, CFI could benefit acceleration and power generation, which are critical for athletic performance. It also has the advantage of being relatively inexpensive and adaptable, as it can be inserted into a variety of shoes and easily replaced. Although it is necessary to investigate muscular activation when wearing CFI, there have been few comprehensive reports on the influence of CFI on muscular activity, subjective comfort, and sports performance [17,25].

Therefore, the purpose of this study is twofold: (1) to investigate whether CFI can improve sports performance; (2) to check if CFI affects muscular activation and wearing comfort during treadmill running. It is hypothesized that the use of CFI will improve sports performance, increase muscular activation, and decrease wearing comfort.

## 2. Materials and Methods

### 2.1. Participants

Thirty healthy young males with shoe sizes between 260 and 270 mm participated in the experiment. All participants were free from any type of back or lower limb pain for at least 6 months, and they were able to complete all of the required tasks. In this study, males with shoe sizes outside of 260 and 270 mm, back or lower limb injuries, heart/lung disease or diabetes, and difficulty in jumping or running were excluded from the experiment. Table 1 summarizes the demographic information of the participants. Written informed consent was obtained from participants. The experimental protocol followed the Helsinki Declaration regarding ethical principles for research involving human subjects [29] and was approved by the Institutional Review Board (IRB NO.: KH2021-140).

### 2.2. Experimental Design

**Figure 1:**
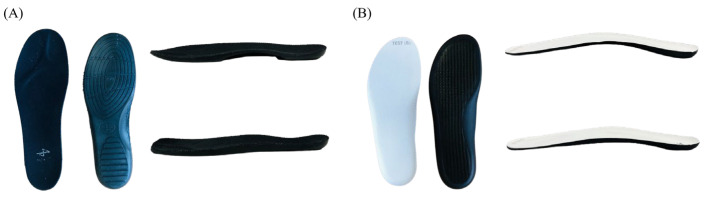
Experimental insoles. (**A**) benchmark commercial insole (COM; SKONO, Norway; MSA5469); (**B**) carbon fiber insole (CFI; YONGJIN FINE CHEMICAL Co. Ltd., Ulsan, Republic of Korea).

A within-subject design was used to investigate the influence of CFI on sports performance, muscle activation, and subjective comfort. Two experimental insoles were tested in random order: benchmark commercial insole (COM) and CFI (Figure 1). The experiment was a randomized within-subject design to minimize variations from individuals and the sequence effect. COM was made of polyurethane foam with an approximate thickness of 0.7 cm towards the front of the insole and 1 cm in the heel. CFI was made of EVA, which included a carbon plate with a thickness of 0.1 cm, and had an approximate thickness of 0.4 cm in the front and 0.4 cm in the heel of the insole. The experiment was conducted with participants’ own sports shoes to better generalize the findings.

### 2.3. Experimental Task

In this study, three aspects of sports performance were examined: the power generation test was composed of a standing long jump (L-jump) and a vertical jump (V-jump); agility was assessed using a 5-10-5-m agility drill (Agility); and speed was assessed using 50-m sprint (Sprint). A standardized procedure was followed to evaluate each sports performance in terms of power generation [8,22,25], agility [21,24], and speed [9,23]. During L-jump, the distance was measured using a steel measuring tape ruler (Model JX-68 5 m, JIUXING, Jiujiang, China). The optical motion capture system was used to measure the jump height during V-jump, and data was recorded with an acquisition frequency of 120 HZ (Model V120, OptiTrack, Corvallis, OR, USA). During the agility drill, a stopwatch and a video recording were used to measure the time (Model XL-013, AnyTime, Woodbury, MN, USA). For the sprint test, completion time was measured with a stopwatch to assess speed.

The effect of CFI on muscle usage was investigated by measuring muscular activation while running on a treadmill (Model S21T, STEX fitness Europe, Mönchengladbach, Germany). The activation of lower extremity muscles was recorded with a sampling frequency of 1000 Hz using surface electromyography sensors (EMG, Bagnoli, Delsys, MA, USA).

### 2.4. Experimental Procedure

**Figure 2:**
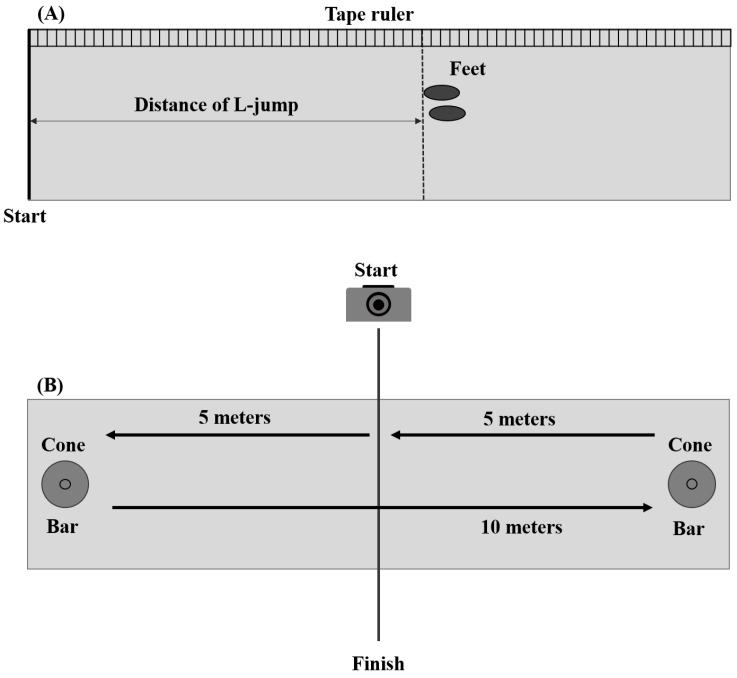
Schematic diagram of sports performance tests ((**A**) L-jump test, (**B**) Agility test).

The experiment consisted of two phases, which were conducted outdoors and indoors, respectively. In the outdoor phase, all participants were asked to perform a stretching and dynamic warm-up for at least 10 min. Considering most of the participants had no experience wearing CFI, they were given sufficient time to familiarize themselves with wearing CFI before a series of sports performance tests. After warm-up and familiarization, participants performed the outdoor experimental task in L-jump, Agility, and Sprint sequence. For the convenience of the experimental setup and test, we conducted the experimental tasks in a consistent sequence instead of a randomized sequence. The sequence of experimental tasks followed the guidelines of the National Strength and Conditioning Association (NSCA) [25,30]. The L-jump consisted of a counter-movement jump with an arm swinging to horizontally jump as far as possible. Participants stood upright with a marked line on the ground, then performed the maximum effort L-jump. For a successful trial, participants were required to land on their feet and were not allowed to move. Performance was assessed as the horizontal distance from the starting line to their shoe heel location (Figure 2A). After finishing the L-jump task, participants performed an Agility composed of three sprints and two cutting movements to change their directions. There were two cones with crossbars where the cutting movement took place, and participants needed to touch the crossbars when changing direction (Figure 2B). Participants in the agility test ran a total of 20 m: 5 m from the start to the cone, 10 m from the cone to the cone, and 5 m from the cone to the finish line. Performance was defined as the time to complete the drill. Lastly, participants performed Sprint to measure their speed. They sprinted from the starting line on the track and stopped after passing the end line marked as the two cones with the bar 50 m from the starting line. The performance outcome was defined as the speed to complete the Sprint.

After finishing all outdoor experimental tasks, the last sports performance test, V-jump, was performed indoors due to the need for an optical motion capture system to accurately measure vertical jump heights. A reflective marker was attached to the tibialis anterior without interfering during the V-jump. The V-jump consisted of a counter-movement jump with an arm swinging to vertically jump as far as possible. Participants stood upright with a marked point on the floor, then performed the maximum effort V-jump. For a successful trial, participants were required to extend their knees and ankles after toe-off, which was visually monitored by the experimenter. Performance was assessed as the height from the reference to the maximum location of the marker, and the reference was defined as the average location of the marker for standing posture for two seconds before the jump. The participants completed three successful trials for each task in each insole condition. If one record deviated from the other two records by more than 10%, the test was considered invalid and the participant was asked to perform the test again to ensure the validity and accuracy of the jump height.

**Figure 3:**
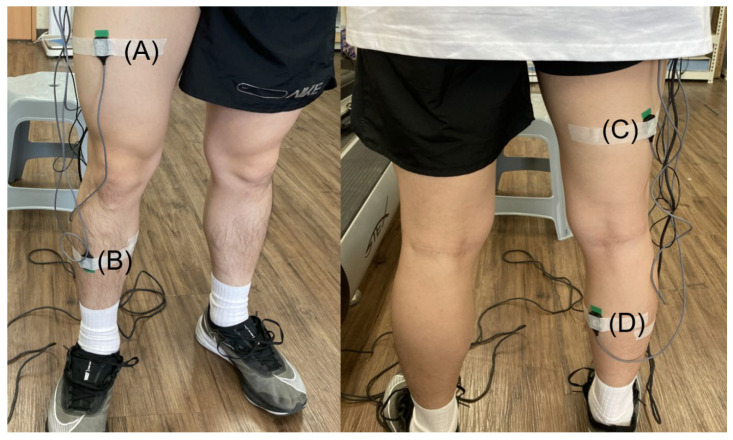
Locations of EMG sensors on the lower extremity ((**A**): Rectus Femoris, (**B**): Tibialis Anterior, (**C**): Biceps Femoris, and (**D**): Gastrocnemius Medialis).

Before the treadmill running task, participants were given at least 10 min to relieve muscular fatigue. Then, the participants’ skin was prepared by removing excessive hair and cleaning with alcohol. Next, four EMG sensors were attached to the skin using adhesive tape and firmly fixed with the strap to minimize potential noise from any detachment or tremble. As shown in Figure 3, four EMG sensors were attached to Rectus Femoris (RF), Tibialis Anterior (TA), Biceps Femoris (BF), and Gastrocnemius Medialis (GM) muscles, which mainly activated muscles during sprinting [31]. The EMG sensors were placed on the middle of the muscle belly, and the longitudinal orientation was parallel to the fascicle orientation [32,33]. Each muscle’s maximum voluntary contraction (MVC) was measured twice, with two minutes of rest between each trial. The maximum MVC trial was used for normalization of the EMG signals.

Participants were instructed to walk at 3 km/h for at least 30 s to familiarize themselves with the task of running on the treadmill. Afterward, they jogged at 6 km/h for at least 30 s and ran at a speed of 10 km/h for 5 min. Participants then ran or walked at their preferred speeds for at least one minute to cool down. After completing a trial run with each insole, participants gave their subjective evaluation of perceived insole stiffness, energy support, overall comfort, and fatigue through a 9-point rating scale. Participants were given at least ten minutes to rest between trials to avoid the fatigue effect.

### 2.5. Data Analysis

Dependent variables were performance measures for each sports task (power generation: distance of L-jump and height of V-jump; agility: completion time of agility test; speed: sprint speed), muscular activation of four muscles, and subjective ratings after treadmill running. The average value of three successful trials during sports was used to determine the effect of CFI. The raw coordinates of a reflective marker during the V-jump were filtered with a cutoff frequency of 10 Hz to smooth the motion trajectory. The EMG data during 5-min running at 10 km/h were rectified, normalized, and smoothed using the root mean square (RMS) filter to perform a linear envelope. For EMG signals, raw signals were filtered at 20–450 Hz to minimize signal noise and smoothed by the RMS filter with a window length of 50 ms.

### 2.6. Statistical Analysis

All data were checked with the Shapiro-Wilk test and box-plot to identify the deviations from normality and detect outliers [34,35]. A paired-samples *t*-test (normally distributed data) or a one-sample Wilcoxon signed-rank test (normality violated) was conducted to statistically evaluate the effects from two different insoles, with the level of significance set to α = 0.05 [36,37,38]. In addition, for parametric data, the effect size was calculated using Cohen’s d which is defined as the difference between two means divided by a standard deviation for the data: Small (d = 0.20), Medium (d = 0.50), Large: (d ≥ 0.80). For nonparametric data, r was calculated by dividing the absolute standardized test statistic by the square root of the number of pairs: Small (r = 0.10), Medium (r = 0.30), Large (r ≥ 0.50) [39,40]. Excel (Microsoft, Redmond, WA, USA) and EMGworks (Delsys, MA, USA) were used to process all data, and SPSS software version 20.0 (IBM Corp., Armonk, NY, USA) was employed to conduct all statistical tests.

## 3. Results

### 3.1. Sports Performance

**Figure 4:**
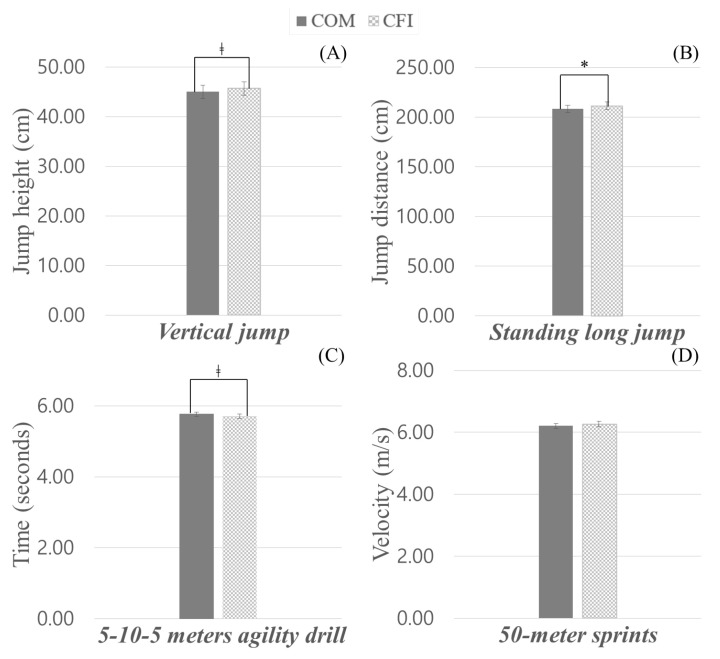
Sports performance measures from the benchmark commercial insole (COM) and carbon fiber insole (CFI). ((**A**) Vertical jump, (**B**) Standing long jump, (**C**) 5-10-5 m agility drill, and (**D**) 50-m sprints). Note. * indicates a statistically significant difference, and ‡ indicates a marginally significant difference.

Parametric comparison results among sports performance from two different insoles (COM, CFI) are presented in Figure 4. CFI wearers showed better performance in jumping. The V-jump height of CFI wearers was averaged at 45.66 cm, marginally higher (*t* = −2.009, *p* = 0.054) than that of COM wearers (44.97 cm). In addition, CFI wearers had significantly greater (*t* = −2.255, *p* = 0.032) L-jump distance than COM wearers (211.27 vs. 208.19 cm). For agility, CFI wearers displayed marginally more agile performance than COM wearers (task completion time: 5.71 s vs. 5.76 s, *t* = 1.712, *p* = 0.098). In Sprint, however, there was no significant difference (*t* = −1.244, *p* = 0.223). Detailed statistical analysis results are summarized in Appendix A (see Table A1).

### 3.2. Muscular Activation

**Figure 5:**
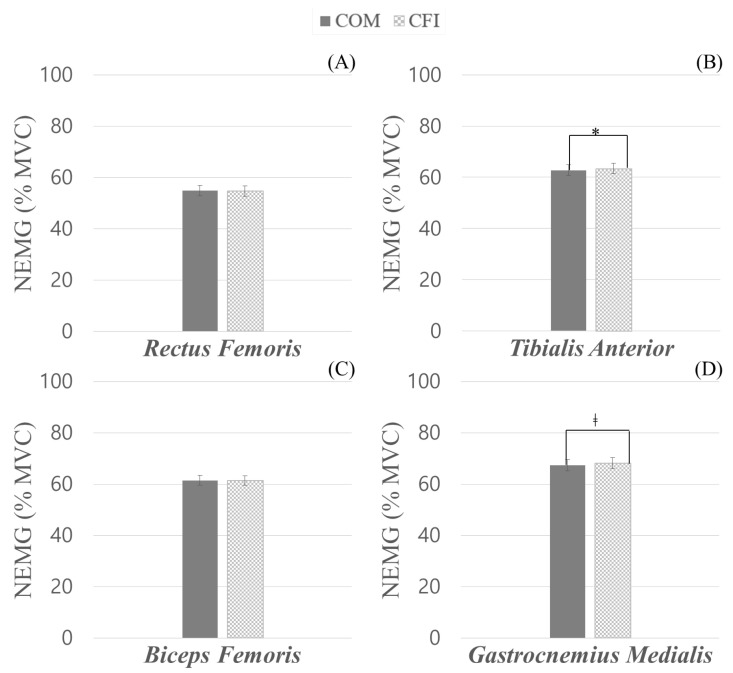
Normalized muscular activation (%) during treadmill running task from the benchmark commercial insole (COM) and carbon fiber insole (CFI). ((**A**) Rectus Femoris, (**B**) Tibialis Anterior, (**C**) Biceps Femoris, and (**D**) Gastrocnemius Medialis). Note. * indicates a statistically significant difference, and ‡ indicates a marginally significant difference.

Figure 5 shows the parametric comparison results on muscular activation from two different insoles. The paired *t*-test revealed significant differences between COM and CFI on activation of TA and GM. However, there was no significant difference between the two different insoles in muscular activation of RF (*t* = 1.428, *p* = 0.164) and BF (*t* = 0.153, *p* = 0.879). When the participants wore CFI, their TA muscle activation was significantly higher (+0.68%; *t* = −2.617, *p* = 0.015) than while wearing COM. In addition, muscular activation of GM while wearing CFI was marginally higher (+0.83%; *t* = −1.902, *p*= 0.067) than when wearing COM. Summary results for comparing muscular activation from two different insoles are summarized in Appendix A (see Table A2).

### 3.3. Subjective Evaluation

**Figure 6:**
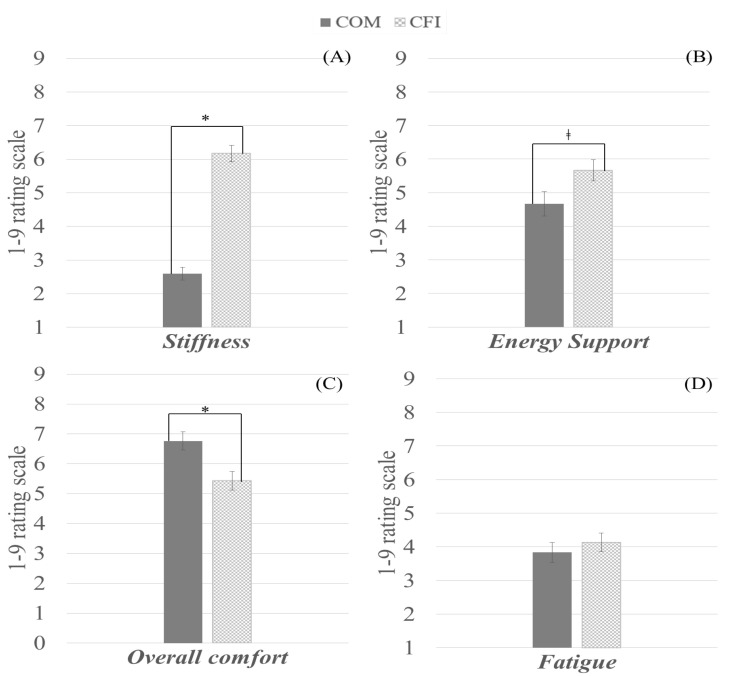
Subjective ratings after treadmill running from the benchmark commercial insole (COM) and carbon fiber insole (CFI). ((**A**) Stiffness, (**B**) Energy support, (**C**) Overall comfort, and (**D**) Fatigue). Note. * indicates a statistically significant difference, and ‡ indicates a marginally significant difference.

Figure 6 presents the non-parametric comparison results on subjective ratings from two different insoles. However, they did not perceive any significant difference in fatigue between the two different insoles (z = −0.861, *p* = 0.389). When the participants wore CFI, they felt that it was significantly stiffer (z = −4.727, *p* < 0.001), provided greater energy support (z = −1.982, *p* = 0.047), but resulted in less comfort (z = 3.048, *p* = 0.002) than when they wore COM. Detailed statistical analysis results are described in Appendix A (see Table A3).

## 4. Discussion

This study aimed to examine the acute effect of CFI on sports performance, lower extremity muscle activity, and subjective comfort. CFI significantly improved sports performance in terms of power generation (~1.5%) and agility (~1%) compared to COM. However, it induced more of TA (~0.7%) and GM (~0.8%) muscles, and was perceived as stiffer and less comfortable while running on a treadmill.

As expected, CFI significantly improved sports performance in terms of power generation and agility compared to COM. In terms of power generation, the average distance of the L-jump and height of the V-jump were improved by about 1.5%. A possible reason for this improvement may be related to energy loss in the MTP joint. While jumping, it has been shown that the MTP joint does not extend until take-off [41]. Since the joint is flexed during stance, there is no energy generated, resulting in lost energy. In terms of sports performance, this energy dissipation appeared to be inefficient. Reduced energy loss in the MTP joint was observed when the participants wore stiffer shoes, which resulted in improved jump performance while wearing these shoes [8]. Thus, wearing CFI, which is stiffer than COM, could improve jump performance by reducing energy loss in the MTP joint. In addition, CFI could help generate power by adding extra gear to the forefoot [15]. An extra gear could increase the moment arm of ground reaction force, resulting in a higher gear ratio. It could induce a slower shortening velocity of the plantar flexor muscles and longer contact time with the forefoot and ground, which could be favorable for force production [16].

There was a marginal improvement (~1%) in agility while wearing CFI. This finding is in line with a previous study that reported that shoes with stiffer plates enhanced agility performance [24]. Even though the underlying reasons for this result remain unclear, one possible hypothesis is that when participants wore CFI, they kept more of their running speed when changing directions. Improvement in agile performance from CFI might be because the carbon fiber plate stiffened the insole to provide energy support, dynamic balance, and ankle joint stability by supporting the ankle. On the other hand, the softer sole provides an unstable support base and decreased somatosensory feedback from the foot’s cutaneous receptors, leading to reduced balance performance than the stiffer sole [42,43]. Thus, it might be one possible reason CFI wearers showed better agile performance than COM wearers. Further research is needed to test this hypothesis with kinematic measurements collected by a motion capture system while performing multi-directional movements.

For sprint running, the effect of footwear stiffness on sprint performance is still being examined among researchers, but the evidence is conflicting. While wearing stiffer shoes, a decrease in sprint time was reported in the previous study [12]. Conversely, stiffer shoes were also reported to have no significant effect on sprint performance [44,45]. This study also did not appear to have any improvements in sprint performance with CFI. It may have originated from factors affecting running performance, such as contact time, ground reaction force, etc. According to a previous study [46], there was an increase in contact time and reduced average ground force application when wearing stiffer shoes, which could have led to decreased sprint performance. The improvement in sprint performance may not have been achieved because the increased contact time due to the use of CFI might offset the improvement in sprint performance obtained by using CFI [16,46]. Furthermore, a higher gear ratio could affect sprint performance. Although several studies have investigated the effect of gear ratio on running performance, it is not well understood. A higher ratio could increase running performance by exerting greater tendon force, leading to greater storage and release of elastic strain energy [47,48]. However, this explanation disregards the possibility that the extra force demands more effort than greater storage and release of energy. During the sprint, larger muscular force is required for propulsion while wearing CFI, and if these are accumulated step by step, there is a possibility that they might have a deleterious effect on the sprint performance. This assumption could be supported by Kovács et al. They reported that a higher gear ratio demanded more muscular effort and concluded that a lower gear ratio could be beneficial for running [49].

Even though the effect of CFI on sports performance has not been examined pertaining to elite athletes, it is possible that athletes could receive more support from CFI. When wearing CFI, sports performance could be improved by adding extra gear to the forefoot [15]. Adding extra gear to the foot could increase the moment arm of ground reaction force, which could lead to a greater moment of the ankle. Thus, wearers should generate more force to respond to increased ankle moment when wearing CFI [50,51]. If the stiffness of CFI is too high for wearers responding to the extra force demands, further increases in stiffness might not help them to enhance their sports performance [49]. Elite athletes, who are generally stronger than normal college students, probably receive more support from CFI. In this study, improvements in sport performance from CFI were minor, as all effect sizes were small to moderate. However, an improvement in sports performance of around 0.36 to 0.63% could change an athlete’s chances of winning a game [52]. Thus, even a small amount of improvement is worthwhile. Power generation and agility performance while wearing CFI was improved by about 1.5% and 1%, respectively. Thus, these changes could be meaningful and practical. However, the effect of CFI on sports performance in elite athletes still needs to be addressed.

Although sports performance can be improved while wearing CFI, when running on a treadmill, muscular activation was significantly higher. When the participants wore CFI, the activation of TA was significantly higher, by 0.7%, with a moderate effect size (Appendix A Table A2). This is because the primary function of the TA muscle is to absorb impact [31,53]; on the other hand, CFI is designed for reducing energy loss to improve sports performance, resulting in impact absorption reduction. Thus, the participants were required to exert the TA muscle more to cushion the impact. Additionally, GM muscle activation was marginally higher by 0.8% (Appendix A Table A2). There are two potential reasons for the increased activation of GM muscle. First, stiffer footwear may change the point of the force application. Previous studies have found that a stiffer shoe’s anterior point of force application was anteriorly moved during the last 25% of the stance phase [54]. It resulted in increasing the participant’s moment arm, which could increase the moment of the ankle joint and the force demand. Because GM muscle is a kind of triceps surae that plays a primary role in absorbing and producing the power of the ankle joint [28], stiffer footwear could lead to greater GM muscle activation. Second, it might come from a higher stiffness of CFI. The insole should be bent during the propulsion phase, but CFI is more difficult to flex than COM [26]. As a result, the participant had to put more effort into bending the CFI, increasing GM muscular activation. Increased muscular activation can lead to localized pain, inflammation, muscle fatigue, and higher oxygen uptake, negatively affecting long-term sports performance [55,56].

Comfort is an essential factor in designing footwear, since uncomfortable footwear can alter gait and lower extremity muscle activity during running, which could affect sports performance as well as the risk of injury [25,57]. In addition, the wearing comfort of the shoes has been identified as a major parameter in considering foot health, energy demand, and muscle strain [22]. In this study, CFI was perceived as uncomfortable compared with COM, since it is significantly stiffer, generating greater muscle activity during the fast run. Furthermore, the effect size of comparing the overall comfort showed a large effect, meaning that there is a practical significance in perceived comfort between CFI and COM (Appendix A Table A3). According to the findings of this study, the use of CFI can improve short-term sports performance and provide energy support, but it also leads to wearing discomfort and increased muscle usage, implying that CFI could negatively influence long-term sports performance. Therefore, CFI should be used appropriately according to the user’s purpose [14,20,58].

Even though our study showed statistically significant differences between CFI and COM in terms of sports performance and muscle activation, as well as subjective comfort, the effect size of the different insoles was small to moderate. This should be understandable, since sports performance is determined by a combination of human characteristics [59,60], equipment [61,62], environment [63,64], and other factors [65,66]. In addition, muscle use during running could be influenced by participants’ running techniques [67,68] and running experience [69]. Thus, deviations from other factors could affect the magnitude of effect size, and changing the insoles only makes small to moderate differences [19].

This study has some limitations. First, although wearing the stiffer CFI improved athletic performance, the optimal stiffness could not be investigated in this study as only two different insoles were available. Second, the duration of the treadmill running (5 min per trial) may be somewhat short compared to some previous studies [11,70]. Thus, the RMS values of the normalized EMG (<70%) in this study were lower than in the previous study [71]. Last but not least, further research should be conducted with insoles of different stiffness to find optimal stiffness for individuals. Even though CFI generally helped wearers perform better in terms of power generation and agility, relative improvements varied from person to person. Furthermore, it remains necessary to examine the long-term effects of CFI, as our study showed potential negative effects of CFI during treadmill running.

## 5. Conclusions

This study investigated the acute effects of CFI on sports performance, lower extremity muscular activation, and subjective comfort. The results showed that, compared to the benchmark insole, CFI significantly improved sports performance in terms of power generation and agility. However, it activated more of the Tibialis Anterior and Gastrocnemius Medialis muscles and was perceived to be stiffer and less comfortable. These findings suggested that CFI can improve sports performance, but it may lead to more muscular activation and subjective discomfort.

## Figures and Tables

**Table 1:** Demographic information of participants.

Characteristics	Mean ± SD (n = 30)
Age (years)	25.4 ± 3.2
Height (cm)	173.2 ± 5.5
Body mass (kg)	69.8 ± 11.3
Shoe size (mm)	266.5 ± 4.6

## Data Availability

The data presented in this study are available on request from the corresponding author.

## Appendix A

**Table A1:** Summary results comparing the sports performance from two different insoles: benchmark commercial insole (COM) versus carbon fiber insole (CFI). (V-jump: Vertical jump, L-jump: Standing long jump).

Tasks		Performance Measurement				Paired *t*-Test	d (Effect Size)
		COM		CFI		*p*	
		Mean ± SD	Range (Min, Max)	Mean ± SD	Range (Min, Max)		
Power test	V-jump (Height, cm)	44.97 ± 7.43	(29.27, 59.58)	45.66 ± 7.27	(27.23, 59.07)	0.054 ^‡^	0.37 (Medium)
	L-jump (Distance, cm)	208.19 ± 19.17	(166.04, 241.34)	211.27 ± 20.75	(156.64, 245.9)	0.032 *	0.41 (Medium)
Agility test	5-10-5 m agility drill (Time, sec)	5.76 ± 0.36	(5.01, 6.64)	5.71 ± 0.36	(5.05, 6.45)	0.098 ^‡^	0.29 (Small)
Speed test	50-m sprints (Speed, m/s)	6.21 ± 0.45	(5.58, 7.43)	6.27 ± 0.49	(5.42, 7.5)	0.223	0.23 (Small)

Remarks: * indicates a statistically significant difference (*p* < 0.05), and ^‡^ indicates a marginally significant difference (0.05 < *p* < 0.10).

**Table A2:** Summary results comparing muscular activation from two different insoles: benchmark commercial insole (COM) versus carbon fiber insole (CFI). (RF: Rectus Femoris, TA: Tibialis Anterior, BF: Biceps Femoris, and GM: Gastrocnemius Medialis).

Localized muscular activation	**Muscles**	**NEMG: % MVC**				**Paired** ***t*-Test**	**d (Effect Size)**
		**COM**		**CFI**		***p***	
		**Mean ±** **SD**	**Range** **(Min, Max)**	**Mean ±** **SD**	**Range** **(Min, Max)**		
	RF	54.85 ± 11.24	(38.63, 86.27)	54.68 ± 11.11	(38.52, 85.7)	0.164	0.27 (Small)
	TA	62.76 ± 11.04	(43.87, 90.75)	63.44 ± 10.90	(45.43, 91.8)	0.015 *	0.50 (Medium)
	BF	61.42 ± 10.24	(43.56, 86.21)	61.40 ± 10.23	(43.34, 86.68)	0.879	0.03 (Small)
	GM	67.33 ± 11.87	(47.22, 93.98)	68.17 ± 11.61	(47.85, 92.1)	0.067 ^‡^	0.35 (Medium)

Remarks: * indicates a statistically significant difference, and ^‡^ indicates a marginally significant difference.

**Table A3:** Summary results comparing subjective ratings from two different insoles: benchmark commercial insole (COM) versus carbon fiber insole (CFI).

Subjective Ratings (1–9 Scale)					Wilcoxon Signed-Rank Test	r (Effect Size)
Items	COM		CFI		*p*	
	Median ± SD	Range (Min, Max)	Median ± SD	Range (Min, Max)		
Stiffness	2.60 ± 1.04	(1, 5)	6.17 ± 1.34	(3, 9)	<0.001 *	0.86 (Large)
Energy Support	4.67 ± 1.97	(2, 9)	5.67 ± 1.71	(3, 8)	0.047 *	0.36 (Medium)
Overall comfort	6.77 ± 1.68	(1, 9)	5.43 ± 1.72	(3, 9)	0.002 *	0.56 (Medium)
Fatigue	3.83 ± 1.60	(2, 7)	4.13 ± 1.55	(2, 7)	0.389	0.16 (Small)

Remarks: * indicates a statistically significant difference.
